# The Blessed Antonio (Patrizi) from Monticiano, Sienna (Italy): Bioanthropological and Palaeohistological Considerations

**DOI:** 10.15388/Amed.2022.29.1.20

**Published:** 2022-06-29

**Authors:** Dario Piombino-Mascali, Albert Zink, Frank Maixner

**Affiliations:** Institute of Biomedical Sciences, Vilnius University, Vilnius, Lithuania; Institute for Mummy Studies, EURAC, Bolzano, Italy; Institute for Mummy Studies, EURAC, Bolzano, Italy

**Keywords:** mummies, relics, biological anthropology, palaeopathology, palaeohistology

## Abstract

**Background:**

A medieval mummy known as the Blessed Antonio (Patrizi) is held in the church of Saints Peter and Paul at Monticiano, Sienna, central Italy.

**Objectives:**

The aim of our investigation was to complete a biological profile of the subject, as well as to assess the impact of deterioration to the concerned remains.

**Methods:**

As a follow-up of our bioanthropological, macroscopic approach, two of the samples taken underwent rehydration, fixation, desiccation, paraffin-embedding, and staining according to standard histological techniques applied to mummified remains.

**Results:**

The body was determined to be that of an adult male, who showed some pathological changes such as dental calculus and what is suspected to be hallux valgus. The overall preservation of a skin sample revealed damage caused by a post-mortem infestation of insects, while a second, inner sample was identified as lung tissue, and revealed a case of anthracosis.

**Conclusions:**

The Blessed Antonio was an adult male, who had poor dental hygiene and was likely exposed to smoke during his lifetime. Damage observed on the remains indicated that a conservation treatment was desirable for the future preservation of the body.

## Santrauka.

**Įvadas:**

Viduramžių mumija, dar žinoma kaip Palaimintasis Antonio (Patrizi), laikoma Šventųjų Petro ir Pauliaus bažnyčioje Monticiano, Sienoje, centrinėje Italijoje.

**Tikslai:**

Mūsų tyrimo tikslas buvo užbaigti biologinį subjekto profilį, taip pat įvertinti šių palaikų būklės pablogėjimą.

**Metodai:**

Tęsiant bioantropologinį ir makroskopinį tyrimą du paimti mėginiai buvo rehidratuoti, fiksuoti, išdžiovinti, įdėti į parafiną ir nudažyti pagal standartinius histologinius metodus, taikomus mumifikuotoms liekanoms.

**Rezultatai:**

Nustatyta, kad tai suaugusio vyro kūnas, turintis tam tikrų patologinių pokyčių, tokių kaip antai dantų akmenys, ir tai, kas, kaip įtariama, yra hallux valgus (iškrypęs pėdos kauliukas). Odos mėginio išsaugojimas atskleidė žalą, kurią po skrodimo sukėlė vabzdžių užkratas, o antrasis vidinis mėginys buvo identifikuotas kaip plaučių audinys ir atskleidė buvus antrakozės atvejį.

**Išvados:**

Palaimintasis Antonio buvo suaugęs vyras, jo burnos higiena buvo labai prasta ir kuris galimai gyvenimo laikotarpiu buvo veikiamas dūmų. Žala, pastebėta ant palaikų, parodė, kad konservavimas buvo reikalingas kūnui išsaugoti ateityje.

## Introduction

**Figure 1.**
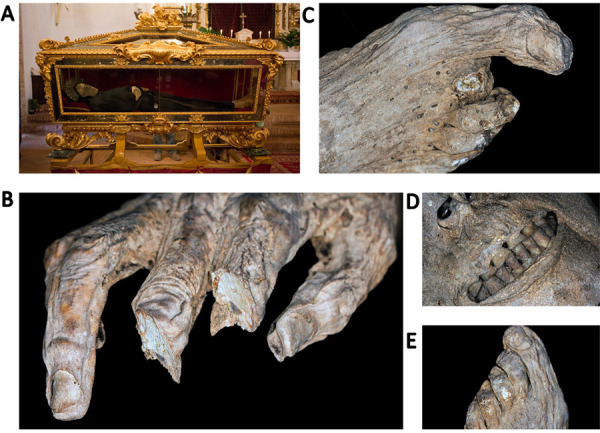
**The spontaneously mummified remains believed to belong to the Blessed Antonio of Monticiano, Sienna.** (A) The body lies in a supine position. (B) Detail of the intentional dissection of the second and third right hand fingers. (C) Detail of the intentional dissection of the second right foot digit. (D) Detail of the visible teeth showing calculus formation. (E) Detail of the right foot revealing mild hallux valgus deformity.

Monticiano is a town in the province of Sienna, Tuscany (central Italy), at 375 meters above sea level. There, the church dedicated to the Saints Peter and Paul houses the mummified remains of a medieval figure known as the Blessed Antonio (Figure 1A). The mummy is an important relic for the local community and, until recently, was displayed during a Catholic procession that took place every ten years. For most of the time, the mummy was located on the main altar of the church, in an opulent, ornamented 17th century urn [1]. In 1995, a transfer of the mummified body into a new urn changed the surrounding environment, leading to a risk of biodeterioration [2]. In order to assess the state of preservation of the mummy, a detailed anthropological inspection had been conducted in October 2011. During this survey, the mummy’s surface had been cleaned, and soft tissue samples had been taken for further microscopic analysis, with the hope of obtaining information on the body’s structural preservation status. This report will therefore provide an overview of the methodologies applied on the sampled material so far. Thereby, the obtained results will be twofold, and will mainly be discussed with regard to the conservation of the mummified human remains.

## Materials and Methods

The mummy of the Blessed underwent a detailed morphological inspection conducted by the authors of this paper in October 2011, which involved macroscopic evaluation of the remains, recording of the anthropological and palaeopathological features, and assessment of the preservation status. During this survey, some sampled material was taken from the remains for further microscopic analysis. The samples were then transferred to the Ancient DNA laboratory of the EURAC – Institute for Mummy Studies, Bolzano (Italy), where the specimens were inventoried and subjected to biomedical investigation. A detailed list of the samples taken during this survey is provided in Table 1. An additional sample consisting of insect remains (# 1529) has already been discussed elsewhere [3]. Soft tissue collected from the mummy was further processed for histological analyses in the aforementioned laboratory. Specifically, small soft tissue fragments (0.5 cm x 0.5 cm) underwent investigation according to the methods described by Mekota and Vermehren [4]. After rehydration via EURAC solution (5 parts glycerol and 5 parts 4% formaldehyde) for 48 h, the samples were fixed for 24 h in 4% formaldehyde, dehydrated, and finally embedded in paraffin blocks. The embedded specimens were cut on a microtome in 4 µm thick sections (Leica, RM2245). The paraffin sections were histochemically counterstained with haematoxylin and eosin (H&E) and Giemsa stain [5].

**Table 1.** List of the Blessed Antonio’s samples

EURAC #	Individual	Sampling site	Sample type	Pieces	Dimension (mm)
1522	Blessed Antonio	Monticiano	Skin tissue	1	45×25
1523	Blessed Antonio	Monticiano	Skin tissue	1	75×20
1524	Blessed Antonio	Monticiano	Textile	1	80×30
1525	Blessed Antonio	Monticiano	Internal tissue	1	105×35
1526	Blessed Antonio	Monticiano	Internal tissue	2	90×30
1527	Blessed Antonio	Monticiano	Internal tissue	1	110×35
1528	Blessed Antonio	Monticiano	Internal tissue	1	65×30

## Results and Discussion

### External Inspection

Upon inspection, the body appeared to be almost complete. It was of a light brown colour and measured 156 cm in length. The remains clearly belonged to a male based on the excellent preservation of the external genitalia. Age at death could only be roughly estimated from the dental wear and lambdoid suture closure, suggesting this was an adult individual (30–50 years old) [6]. The remains lay in a supine position, with the arms crossed over the abdomen and the legs extended. Flattening of the posterior surface of the corpse due to premature deposition on a surface was observed. No signs of evisceration or defleshing were identified, indicating that the process of mummification was spontaneous. This is consistent with many mummies of Saints coming from different areas of the country, which can be considered naturally, or spontaneously preserved [7]. Desiccation took place for natural reasons due to the particular environmental conditions of the tomb, of its micro-climate and, more generally, of the local climate. In many such cases, a pristine mummification was achieved, whereas in other instances an incomplete mummification occurred following an initial and partial decay [8]. Regarding central Italy, additional examples of an excellent, well-documented spontaneous preservation can be found in Tuscany, such as the bodies of Saint Davinus of Armenia, Saint Zita of Lucca, the Blessed Libertesca of Buriano (a mummy deprived of her head), Saint Agnes of Montepulciano, and Saint Antoninus of Florence; furthermore, such type of remains is also common in Umbria, where the Blessed Ubald of Gubbio, Antonia of Florence, and Vincent of Aquila were laid to rest after death [8-11]. As far as the completeness of the body is concerned, however, lack of soft tissue was visible around some areas, such as both scapulae, the right back, the left flank, and the left and dorsal neck. The second and third right hand fingers, as well as the second right foot digit, had been intentionally dissected with the aim of creating relics (Figure 1B & Figure 1C) [1]. Furthermore, a large number of puparia appeared to be located in gravity-dependent, degraded areas. Scattered holes consistent with dermestid activity were also visible on the skin [3]. From a palaeopathological viewpoint, the retracted lips allowed for the identification of a remarkable dental attrition that affected the upper incisors, as well as calculus formation on the lower incisors, suggestive of poor dental hygiene (Figure 1D). Lastly, the feet of the decedent suggested the presence of a mild forefoot deformity known as hallux valgus, possibly caused by the use of pointed shoes (Figure 1E) [12].

### Palaeohistology

**Figure 2.**
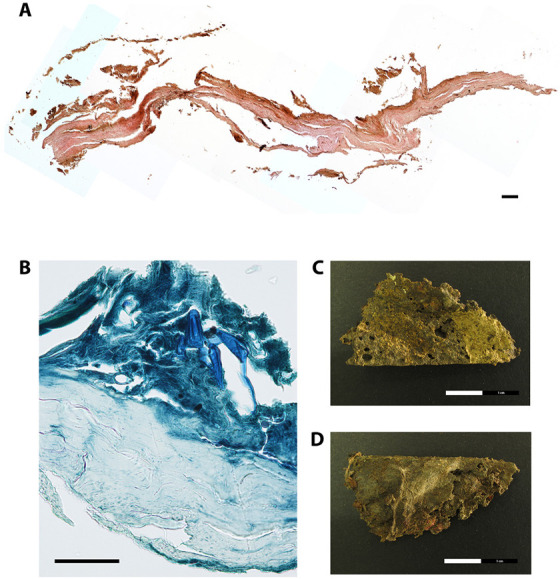
**Paraffin cross-section of skin tissue of the thoracic back side of the mummified individual (EURAC-ID 1522).** (A) Overview picture of the skin layers (consisting of 10 merged single pictures), H&E stain, bar=50µm. (B) Detailed view of the skin tissue layers, Giemsa stain, bar=50µm. (C) Skin tissue sample used for histological analysis, front side view, bar=2cm. (D) Skin tissue sample used for histological analysis, back side view, bar=2cm.

**Figure 3.**
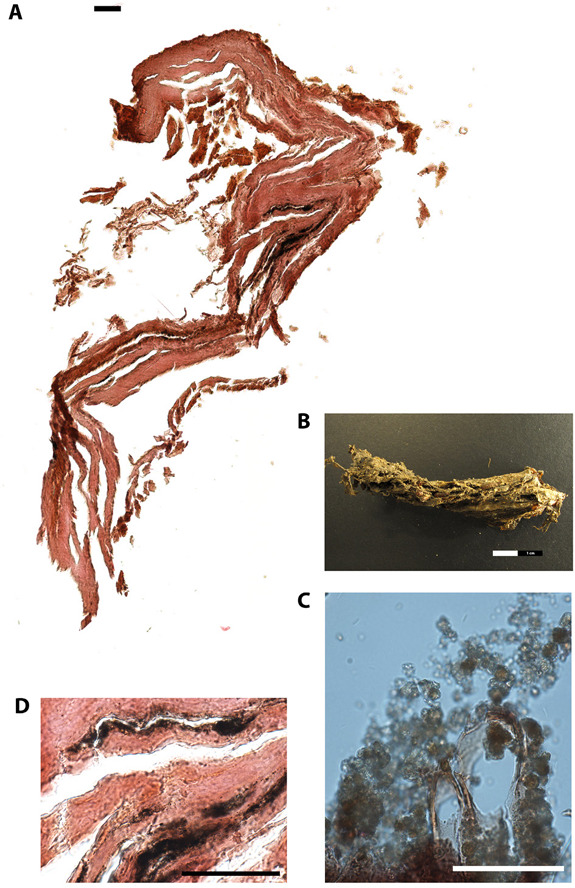
**Paraffin cross-section of internal tissue of the mummified individual (EURAC-ID 1526).** (A) Overview picture of the internal tissue (consisting of 6 merged single pictures), H&E stain, bar=50µm. (B) Internal tissue sample used for histological analysis, front side view, bar=2cm. (C) Detailed view of the internal tissue sample, H&E stain, bar=50µm. (D) Detailed view of the internal tissue sample, Giemsa stain, bar=50µm.

As previously mentioned, selected soft tissue material retrieved during the survey was subjected to palaeohistological analysis to first assess the overall structural preservation and the degree of microbial contamination (Table 2). In addition, morphological details in the tissue sections, possible pathological alterations of the tissue material, and the staining quality were also recorded. The overall structure of the skin tissue was not well-preserved, at least in the sampled area (thoracic back side) (# 1522) (Figure 2A). The staining quality was poor. The connective tissue was partly disrupted and structural details were hardly addressable. Only the dermis could be recognized. The skin tissue lacked an intact epidermis and cell nuclei. These observations are typical for mummified human remains and were reported in numerous studies before (e.g., [7, 13, 14]). A subcutaneous fat layer was missing. The upper brownish part possibly resembles remnants of the epidermis (Figure 2A). A clear identification, however, is difficult since structural details such as melanosomes embedded in keratinocytes were missing. The Giemsa-stained sections revealed no actual colonisation of the tissue by microorganisms. In the upper skin layers, however, inclusions were visible, possibly displaying remnants of insects, most presumably larvae (Figure 2B). This first assumption that the soft tissue was colonised post-mortem by insects is further supported by several insect holes clearly visible by eye in the skin tissue specimen (Figure 2C & Figure 2D). Post-mortem soft tissue destruction by insects is a well-known decay process for mummified individuals, as beetle and mite fragments can be often found microscopically in tissue cavities [15]. In this specific situation, a previous identification of puparia suggested that an initial colonisation by a muscid fly called *Ophyra capensis* took place, and it likely stopped when the body reached a critical level of desiccation. Nevertheless, the puncture damage observed on the mummy could be associated to later dermestid beetles, which feed on dry material [3]. The second histologically investigated specimen (# 1526) had been retrieved from the inner thoracic part of the mummified body and it was believed to represent pulmonary soft tissue remains. Initially, however, the microscopic overview picture revealed no lung tissue characteristic details such as thin-walled alveoli composed of a single layer of squamous epithelium [5]. In contrast to recent lung tissue alveoli, which are separated by thin layer of connective tissue and numerous capillaries, thick layers of connective tissue are more indicative for rigid internal tissue structures such as the trachea or the oesophagus (Figure 3A). The first indications for a tubular tissue system were further supported by the round and stretched-out structure of the tissue specimen (Figure 3B). A detailed histological view of the internal tissue specimen revealed, however, black incrustations generally classifiable as anthracosis of the lung tissue (Figure 3C). In this scenario, inhaled coal particles became phagocytized by macrophages but not eliminated. These black particles accumulated in the lung tissue, causing chronic irritation to the alveolar walls [16]. Within mummy studies, similar findings were also reported from Egypt, the Canary Islands, Italy, and the Arctic [17-21]. These subjects may have been exposed to smoke inhalation, which likely reflects poor ventilation in rooms with open hearths or even participation in rituals [7, 22]. Taken together, the presence of black deposits in this case, coupled with the randomly observable tree-like structures evocative for pulmonary alveoli, strongly suggest that the analysed specimen is from lung tissue.

**Table 2.** List of samples used for microscopic analysis

EURAC #	Individual	Sampling site	Sample type	Histology H&E	Histology Giemsa
1522	Blessed Antonio	Monticiano	Skin tissue	**X**	**X**
1526	Blessed Antonio	Monticiano	Internal tissue	**X**	**X**

## Conclusions

The present results of the microscopic analysis provide insights into the current state of preservation of the tissue material from the mummified individual known as the Blessed Antonio of Monticiano. Desiccated tissue samples display a quite variable preservation status considering the histological criteria of the overall structural preservation and the staining quality. The overall structure of the skin tissue taken from a degraded area is not well-preserved and displays clear signs of post-mortem degradation caused by insects. The second analysed internal tissue specimen could be identified as lung tissue based on pulmonary alveolus-like structures and the presence of black deposits, which are consistent with a case of anthracosis determined by smoke inhalation. Future radiological studies would be a valuable addition to the overall analysis of these remains.
